# Accuracy of Demirjian's and Cameriere's Methods for Age Estimation in 6- to 10-Year-Old Iranian Children Using Panoramic Radiographs

**DOI:** 10.1155/2022/4948210

**Published:** 2022-08-23

**Authors:** S. Milani, M. Shahrabi, H. B Fakhar, S. Parvar, M. Abdolahzadeh

**Affiliations:** ^1^Department of Pediatric Dentistry, Tehran University of Medical Sciences, Tehran, Iran; ^2^Department of Maxillofacial Radiology, Faculty of Dentistry, Tehran University of Medical Sciences, Tehran, Iran

## Abstract

**Objective:**

This study assessed the accuracy of Demirjian's and Cameriere's methods for age estimation in Iranian children using panoramic radiographs.

**Materials and Methods:**

This cross-sectional study evaluated 212 panoramic radiographs of 6- to 10-year-old children retrieved from the archives of an oral and maxillofacial radiology department from 2011 to 2017. The chronological age of children at the time of radiography was determined by subtracting the date of radiography from their birth date. The developmental stage of 7 permanent left mandibular teeth was determined according to Demirjian's method. The stage of dental maturation was determined according to Cameriere's method by using the normalized values for 7 permanent left mandibular teeth and the number of teeth with complete root development. The error value of the two methods was calculated by comparing them with the actual chronological age of male and female children, and the absolute error values of the two methods were compared with paired *t*-tests.

**Results:**

The mean error value of Demirjian's and Cameriere's methods was found to be 0.84 and −0.06 in girls and 0.93 and 0.04 in boys, respectively. Significant differences were noted in the absolute error of the two methods compared with the chronological age of male and female children (both *Ps* < 0.001).

**Conclusion:**

In conclusion, this study indicated that Cameriere's method was more accurate than Demirjian's method for age estimation in Iranian children.

## 1. Introduction

Estimation of the chronological age based on dental age is valuable in forensic medicine, archeology, and biology [1]. Precise age determination is highly important in both living and deceased individuals particularly in children and adolescents [2]. In living individuals, forensic approaches may be required for age determination based on diagnostic evidence in the absence of reliable documentation [3].

Although cementum and dentin are used for age estimation in a corpse, clinical examination and radiography are highly important in living individuals [4]. Root development, periodontium, pulp size/tooth size ratio, and crown size/root size ratio of permanent teeth can all be evaluated on radiographs. The results of the methods such as assessment of the morphology of primary and permanent dentition [5], degree of calcification of tooth structure [6], biochemical findings in dental hard tissue, and age-related changes in the human genome [7] have been controversial regarding age estimation.

On the other hand, the human dentition and maturation process is commonly used for age estimation in humans due to their lower dependence on environmental factors [2, 3]. The more accurate methods of age estimation are based on radiographic examination of the pattern of development in the permanent teeth [3]. In forensic dentistry, dental age estimation methods should yield accurate, reliable, and comparable results. Also, the results should be as close as possible to the actual chronological age of individuals [8]. Due to the differences in dental development and maturation process in different races and geographical areas and the existing variations in the results of radiographic modalities for age estimation, independent studies on each racial group are warranted [1].

Skeletal age is the most commonly used and most accurate method of age estimation in forensic medicine, and the hand wrist and phalanges are most commonly used for this purpose [9]. Around 30 small bones can be evaluated at the same time on one radiograph. The diagnosis cannot be made based on the observation of one bone alone, but the determination of the exact developmental age is possible based on the observation of the level of development of all bones [10]. Also, assessment of the occurrence of specific skeletal events in this region indicates the remaining time until the growth spurt and enables the prediction of speed and the residual amount of growth in an individual [11]. However, this method is difficult and time-consuming for dental clinicians.

Thus, they are more interested in using dental age for this purpose.

Several methods are used for the determination of dental age. Dental age can be estimated based on the time of eruption of the teeth, which is highly influenced by the environmental parameters. Alternatively, it can be estimated based on the degree of root resorption of the primary teeth, which is only useful during a specific period. Radiographic assessment of the crown and root development of the teeth has shown the most accurate results for age estimation [12]. Evidence shows that dental age is more in agreement with chronological age rather than showing the actual developmental status of an individual [13].

Demirjian's method is the most accepted method of dental age estimation worldwide [14], which has been used in different races [5]. In this method, the tooth buds of 7 permanent left mandibular teeth on the radiographs are evaluated. The original study by Demirjian was conducted on French-Canadian children [15]. The accuracy and applicability of Demirjian's method for age estimation have been evaluated in different countries, yielding controversial results [16]. Cameriere's method is also used for age estimation, which is based on the correlation between age and open-apex teeth in European populations [17]. On the other hand, panoramic radiography is routinely requested for many dental procedures such as orthodontic treatment. According to Demirjian et al. [5], panoramic radiographs can be used for the assessment of tooth calcification stages and for the subsequent determination of dental age.

Considering all the above, this study aimed to assess the accuracy of Demirjian's and Cameriere's methods for age estimation in 6- to 10-year-old Iranian children by using panoramic radiographs.

## 2. Materials and Methods

This descriptive, cross-sectional study was conducted using panoramic radiographs on children between 6 and 10 years retrieved from the archives of the Oral and Maxillofacial Radiology Department of School of Dentistry, Tehran University of Medical Sciences. The study protocol was approved by the ethics committee of the Tehran University of Medical Sciences (IR.TUMS.DENTISTRY.REC.1397.094).

The sample size was calculated to be 104 for each gender (a total of 208). The inclusion criteria were panoramic radiographs of boys and girls between 6 and 10 years with all permanent teeth in the process of development in the left quadrant of the mandible. The panoramic radiographs were taken for 6 years from 2011 to 2017. Inclusion criteria included children who had all the evolving permanent teeth of the mandibular left quadrant. The chronological age of children at the time of radiography was determined by subtracting the date of radiography from their birth date. The radiographs had been obtained for diagnostic and therapeutic purposes and were not related to this study. Also, the parents or legal guardians of children had consented to the use of panoramic radiographs of their children for research purposes.

For age estimation by Demirjian's method [5], 7 permanent left mandibular teeth were evaluated. The developmental stage of each tooth was determined according to Demirjian's method.

**Figure 1:**
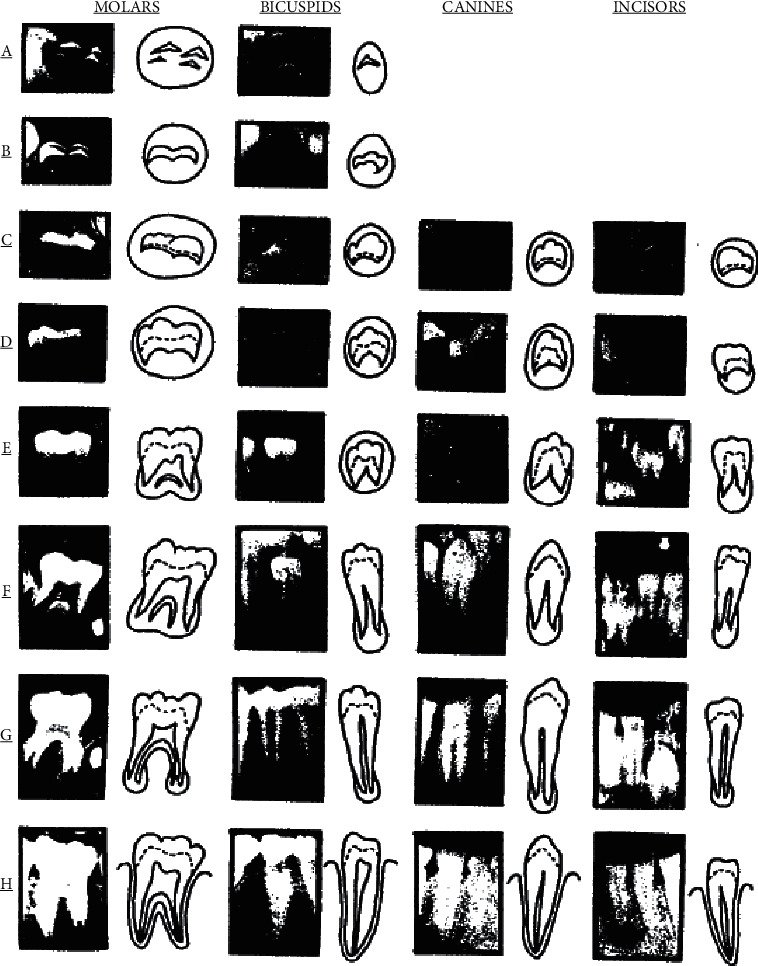
Developmental stages of permanent teeth according to Demirjian's method [18].

The process followed for age estimation are as follows:

- Permanent left mandibular teeth were ranked as follows: second molar, first molar, second premolar, first premolar, canine, lateral incisor, and central incisor.
- All teeth were coded A to H as shown in Figure 1 using the available instructions. In the instructions, each dental stage had 1, 2, or 3 characteristics. If a stage had only one defining characteristic, the tooth should meet the respective characteristic in order to be assigned to a respective stage. If a stage had two characteristics, only the presence of the first characteristic would suffice. If a stage has 3 characteristics, the first two characteristics should be met in order for a tooth to be assigned to the respective stage. Teeth assigned to a particular stage should meet all the characteristics of the previous stage(s) as well. For borderline cases, the lower (earlier) stage was considered for the respective tooth.
- A magnifier was not used for the assessment of apex closure, and staging was performed by the naked eye.
- Crown length was defined as the maximum distance between the highest cusp tip to the cementoenamel junction. In cases where the buccal and lingual cusp tips were not at the same level, their midpoint was considered the highest point.

The developmental stages observed are as follows:

- In both single-rooted and double-rooted teeth, the onset of calcification was seen at the peak of the tooth bud in the form of a cone or an inverted cone. The calcified points were not connected.
- Connection of calcified points led to the development of cusp(s) such that the occlusal surface of the tooth was outlined.
- (a) Enamel formation of the occlusal surface was completed, and it extended towards the cervical region; (b) dentin formation was initiated; and (c) the outline of the pulp chamber was seen in the form of a curve at the occlusal border.
- (a) Formation of the crown was completed to the level of the cementoenamel junction; (b) the superior border of the pulp chamber in single-rooted teeth was seen in the form of a curve, which was concave towards the cervical region. Pulp horns, if present, were seen in the form of an umbrella tip. In molars, the pulp chamber had a trapezoidal form, and (c) the initiation of the root formation was seen in the form of a spicule.
- Single-rooted teeth: (a) pulp chamber walls formed straight lines, which were interrupted by the pulp horns, and had become larger compared with the previous stage; (b) the root length was shorter than the crown length. Molars: (a) primary formation of root bifurcation was noted in the form of a calcified or semilunar shape; (b) the root length was shorter than the crown length.
- Single-rooted teeth: (a) pulp chamber walls formed separate triangles; (b) the root length was equal to or longer than the crown length. Molars: (a) the calcified bifurcation area had grown downward, conferring a more distinct shape to the roots with a funnel-shaped apical region; (b) root length was equal to or longer than the crown length.
- Root canal walls were parallel, and part of the apex was still open (the distal root of molar teeth)
- (a) The apex of the root canal was completely closed (the distal root of molars); (b) the periodontal membrane had a uniform width around the root and apex.

Each tooth was then assigned to a developmental stage, and the respective tables by Demirjian were used for scoring each stage [5]. For example, the first molar tooth of a boy in stage E was allocated a score of 9.6. The scores of all 7 teeth were summed to calculate the maturity score. The maturity scores were placed in the respective tables to determine the equivalent age [5]. The maturity score was converted to dental age using the respective tables provided by Demirjian et al. [5]. For instance, a score of 40 for a boy indicated a chronological age of 6.9 years.

**Figure 2:**
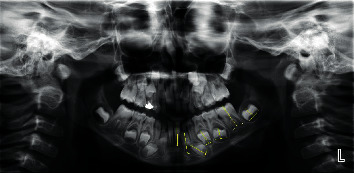
Application of Cameriere's method for dental age estimation according to a panoramic radiograph.

For age estimation using Cameriere's method, 7 permanent mandibular left teeth were used [17]. For this purpose, the number of teeth with complete root development and closed apices was recorded (N0). Teeth with inadequate root development and open-apex teeth were also identified. For single-rooted teeth, the distance between the internal surfaces of the open apices (Ai, *i* = 1–5) and in double-rooted teeth, (Ai, *i* = 6.7) the total distance between the internal surfaces of the two open apices was calculated (Figure 2).

In order to control the possible difference in image magnifications and angles on radiographs, the sizes were normalized by dividing them by the tooth length (Li, *i*: 1–7) (Xi = Ai/Li, *i* = 1–7). Finally, dental maturation was calculated by using the normalized values for the 7 permanent left mandibular teeth. The sum of these values (S) and the number of teeth with complete root development (N0) were calculated. All calculations were performed by one operator, and Cameriere formula was calculated as

(1) $$\begin{array}{c} \text{Age}=8.791+0.375g+1.631X5+0.674N0-1.034s-0.176s, \end{array}$$

where *g* is the variable equal to 1 in boys and 0 in girls, *X*5 is the maturity score of the second premolar (X5 = A5/L5), N0 is the number of teeth with closed apex and developed root, and S is the sum of the maturity scores of all open-apex teeth.

Cronbach's alpha for the assessment of the reliability of dental age calculations by Demirjian's method was calculated to be 0.728 in girls and 0.839 in boys. Cronbach's alpha for the assessment of dental age calculations by Cameriere's method was calculated to be 0.758 in girls and 0.855 in boys.

Data were analyzed using SPSS version 25. The mean and standard deviation of chronological age and dental age according to the Demirjian's and Cameriere's methods were reported separately for girls and boys. Also, the error of dental age estimation by the aforementioned methods compared with the chronological age was calculated and reported separately for males and females.

Using paired means power analysis option in PASS11 software and considering *α* = 0.05 and *β* = 0.2, the mean difference obtained is equal to 1 year and the mean standard deviation is 3.6 years according to the results of a study by Javadinejad et al. 2015 [15].

The absolute error of the two methods was also calculated and compared separately in boys and girls using paired *t*-tests. The level of significance was set at 0.05.

## 3. Results

Table 1 presents the measures of central dispersion for the chronological age and dental age according to Demirjian's and Cameriere's methods in girls and boys.  Table 2 presents the measures of central dispersion for the mean error of the two age estimation methods in girls and boys. In girls, the mean error of Demirjian's and Cameriere's methods for chronological age estimation was 0.84 ± 1.03 and −0.06 ± 0.93, respectively, while the mean absolute errors for boys were 1.05 ± 0.82 and 0.76 ± 0.53, respectively. According to the paired *t*-test, significant differences were noted in absolute error values of age estimation according to Demirjian's and Cameriere's methods (a mean difference of 0.29, *P*<0.01), and Cameriere's method in girls gave a more accurate estimation, and the values were closer to the actual chronological age compared with Demirjian's method.

In boys, the mean error of Demirjian's and Cameriere's methods for chronological age estimation was 0.93 ± 0.87 and −0.04 ± 0.83, respectively, while the mean absolute errors were 1.05 ± 0.72 and 0.64 ± 0.53, respectively. According to the paired *t*-test, significant differences were noted in absolute error values of age estimation according to Demirjian's and Cameriere's methods (a mean difference of 0.41, *P*<0.001), and Cameriere's method in boys gave a more accurate estimation, and the values were closer to the actual chronological age compared with Demirjian's method.

## 4. Discussion

Age determination is a major concern in medical and legal procedures. The present study assessed the accuracy of Demirjian's and Cameriere's methods to determine the chronological age of 6- to 10-year-old Iranian children using panoramic radiographs. The results showed that Demirjian's method overestimated the chronological age of girls by 0.84 years, while Cameriere's method underestimated the age of girls by 0.06 years. Accordingly, Cameriere's method was more accurate than Demirjian's method for age estimation in girls. On the other hand, Demirjian's method overestimated the age of boys by 0.93 years and Cameriere's method overestimated the age of boys by 0.04 years; these results indicated higher accuracy of Cameriere's method for age estimation in boys.

Similar to the majority of previous researches [19–21] Tunc et al. and Koyuturk et al. [22] reported that the chronological age of Turkish boys was 0.36–1.43 years and that of girls was 0.5–1.44 years ahead of the age estimated by Demirjian's method. Their results agreed with the present findings regarding the accuracy of Demirjian's method. Also, Prabhakar et al. [23] reported that Indian boys were 1.2 years and girls were 0.9 years ahead of the estimated age by Demirjian's method, which was in accordance with the present findings.

Hegde and Sood [24] evaluated 6- to 13-year-old Belgian children and found that the difference between dental age and chronological age according to Demirjian's method was 0.14 years for boys and 0.04 years for girls, such that Demirjian's method overestimated the chronological age of both girls and boys; the different values reported in their study were smaller than those found in the present study. Controversy in the results of the studies may be due to racial differences and due to the larger age range of Belgian children. Chaillet et al. [25] reported that Demirjian's method overestimated the age of 6- to 15-year-olds, irrespective of gender, which was in line with the present findings. Lee et al. [26] evaluated 1483 Korean children between 3 and 16 years and showed that Demirjian's method overestimated the age by 0.28 years in boys and 0.33 years in girls. Their results were in accordance with the present findings; however, the difference in estimated values and the actual age in their study were smaller than the corresponding values in the present study, which may be due to racial differences or differences in sample size and age range of children in the two studies.

Altan et al. [27] evaluated 4- to 15.99-year-old Turkish children and reported that Demirjian's method overestimated the age by 0.832 years in girls and by 0.923 years in boys, which was in line with the present results. Galić et al. [28] evaluated 6- to 13-year-old Bosnian and Herzegovinian children and reported that Cameriere's method overestimated the age by 0.09 years in females and underestimated the age by 0.02 years in boys. Their results were different from the present findings obtained by Cameriere's method, which may be due to racial differences and the differences in the age range of children. Fernandes et al. [29] estimated the age of 5- to 15-year-old Brazilian children by Cameriere's method and reported that this method overestimated the age of children, which was in line with the present findings in boys but different from that in girls. Variations in the results may be attributed to racial differences and the different age ranges of children in the two studies. Apaydin and Yasar [30] evaluated the panoramic radiographs of 330 Turkish children between 5 and 15.9 years and reported that Demirjian's method overestimated the age by 0.304 years, while Cameriere's method underestimated the age by 0.58 years. Their findings regarding the accuracy of Demirjian's and Cameriere's methods in girls were in line with the present results; however, their results regarding Cameriere's method were different from the present findings in boys. Moreover, they reported that the error rate was lower in Demirjian's method, which was different from the present findings. Variations in the results may be due to racial differences, which can cause different growth patterns, as well as differences in sample size and age range of children.

Wolf et al. [31] evaluated 479 panoramic radiographs of 6- to 14-year-old German children and reported that the Demirjian's method overestimated the age in both boys and girls. They added that Cameriere's method overestimated the age of 6- to 11-year-old boys and 6- to 10-year-old girls. Their results were generally in agreement with the present findings except that Cameriere's method underestimated the age of girls in the present study. However, they concluded that Demirjian's method was more suitable for age estimation, which was different from the present results probably due to racial and methodological differences since they assessed the right mandibular teeth. A meta-analysis conducted on Australian, Belgian, Canadian, English, Finnish, French, South Korean, and Swedish children found no significant difference in the timing of tooth bud developmental stages among them and reported that Demirjian's method was accurate enough for age estimation in all of them [32].

The present study showed that the mean absolute error for Demirjian's and Cameriere's methods was 1.05 and 0.76, respectively, in girls, and 1.05 and 0.64, respectively, in boys. The paired *t*-test revealed a significant difference between the two methods in this respect. In a study conducted on Bosnian and Herzegovinian children, the absolute accuracy of Cameriere's method was 0.53 in girls and 0.55 in boys, which were lower than the values in the present study. This difference may be attributed to differences in sample size and racial differences among children. In general, variations in the results reported in the literature can be due to the different methodologies and racial and sample size differences, variations in the age range of study populations, statistical methods, environmental, and nutritional parameters, and variations in the expertise of the operators, and different socioeconomic classes [33]. Despite the fact that racial, environmental, and nutritional differences may accelerate or decelerate the growth spurt, the difference in chronological and dental age decreases as individuals age, and the reasons for less variability in dental age are not fully understood. A possible reason is that the development of all the deciduous dentition and part of the permanent dentition takes place before birth in a protected environment, whereas skeletal growth and development, even though having a strong genetic basis, is exposed for an increasing length of time to external factors such as variations in nutrition, socioeconomic status, and possibly climate [34].

This study was a single-center study and evaluated a limited age group; thus, the results may not be generalizable to the entire population of Iran. Considering the limited number of studies on the accuracy of age estimation by Demirjian's and Cameriere's methods in the Iranian population, further multicenter studies are required on different ethnic and age groups in Iran. Also, tables specific to the Iranian population should be compiled for conversion of dental age to chronological age.

## 5. Conclusion

Demirjian's method overestimated the chronological age of 6- to10-year-old male and female Iranian children while Cameriere's method underestimated the chronological age of girls and overestimated that of boys. Overall, Cameriere's method was more accurate for age estimation in Iranian children.

## Figures and Tables

**Table 1:** Measures of central dispersion for the chronological age and dental age of girls and boys according to Demirjian's and Cameriere's methods.

Gender	Parameter	Chronological age (in years)	Dental age according to Demirjian's method (in years)	Dental age according to Cameriere's method (in years)
Girls	Mean	8.43	9.27	8.38
	Std. deviation	0.89	1.3	1.19
	Minimum	6.18	7.4	5.74
	Maximum	9.96	13.1	11.16
	Number	107	107	107

Boys	Mean	8.42	9.35	8.46
	Std. deviation	1.07	1.26	1.25
	Minimum	6.08	7.1	5.49
	Maximum	10.0	11.7	10.72
	Number	105	105	105

**Table 2:** Measures of central dispersion for the mean error of the two age estimation methods in girls and boys.

Gender	Parameter	The error of Demirjian's method	The error of Cameriere's method	The absolute error of Demirjian's method	The absolute error of Cameriere's method
Girls	Mean	0.84	−0.06	1.05	0.76
	Std. deviation	1.03	0.93	0.82	0.53
	Minimum	−1.54	−1.92	0	0.02
	Maximum	4.23	2.83	4.23	2.83
	Number	107	107	107	107

Boys	Mean	0.93	0.04	1.05	0.64
	Std. deviation	0.87	0.83	0.72	0.53
	Minimum	−1.08	−2.04	0.01	0
	Maximum	3.9	2.97	3.9	2.97
	Number	105	105	105	105

## Data Availability

The collection of data to support the results of this article is available upon request to the corresponding author.
